# Reclassification of TCGA Diffuse Glioma Profiles Linked to Transcriptomic, Epigenetic, Genomic and Clinical Data, According to the 2021 WHO CNS Tumor Classification

**DOI:** 10.3390/ijms24010157

**Published:** 2022-12-21

**Authors:** Galina Zakharova, Victor Efimov, Mikhail Raevskiy, Pavel Rumiantsev, Alexander Gudkov, Oksana Belogurova-Ovchinnikova, Maksim Sorokin, Anton Buzdin

**Affiliations:** 1 World-Class Research Center “Digital Biodesign and Personalized Healthcare”, Sechenov First Moscow State Medical University, 119048 Moscow, Russia; 2 Multidisciplinary Medical Center, Group of Clinics, 194044 Saint-Petersburg, Russia; 3 Moscow Institute of Physics and Technology, 141701 Dolgoprudny, Russia; 4 Shemyakin-Ovchinnikov Institute of Bioorganic Chemistry, 117997 Moscow, Russia; 5 PathoBiology Group, European Organization for Research and Treatment of Cancer (EORTC), 1200 Brussels, Belgium

**Keywords:** cancer biomarkers, genomics, transcriptomics, epigenetic profiles, TCGA, The Cancer Genome Atlas, WHO CNS5, classification of CNS tumors, adult-type diffuse gliomas, glioblastoma

## Abstract

In 2021, the fifth edition of the WHO classification of tumors of the central nervous system (WHO CNS5) was published. Molecular features of tumors were directly incorporated into the diagnostic decision tree, thus affecting both the typing and staging of the tumor. It has changed the traditional approach, based solely on histopathological classification. The Cancer Genome Atlas project (TCGA) is one of the main sources of molecular information about gliomas, including clinically annotated transcriptomic and genomic profiles. Although TCGA itself has played a pivotal role in developing the WHO CNS5 classification, its proprietary databases still retain outdated diagnoses which frequently appear incorrect and misleading according to the WHO CNS5 standards. We aimed to define the up-to-date annotations for gliomas from TCGA’s database that other scientists can use in their research. Based on WHO CNS5 guidelines, we developed an algorithm for the reclassification of TCGA glioma samples by molecular features. We updated tumor type and diagnosis for 828 out of a total of 1122 TCGA glioma cases, after which available transcriptomic and methylation data showed clustering features more consistent with the updated grouping. We also observed better stratification by overall survival for the updated diagnoses, yet WHO grade 3 *IDH*-mutant oligodendrogliomas and astrocytomas are still indistinguishable. We also detected altered performance in the previous diagnostic transcriptomic molecular biomarkers (expression of *SPRY1*, *CRNDE* and *FREM2* genes and FREM2 molecular pathway) and prognostic gene signature (*FN1*, *ITGA5*, *OSMR*, and *NGFR*) after reclassification. Thus, we conclude that further efforts are needed to reconsider glioma molecular biomarkers.

## 1. Introduction

Diffusely infiltrating gliomas are the most common primary malignant brain tumors in adults [1]. They include astrocytomas and oligodendrogliomas that grow from different types of glial cells (astrocytes and oligodendroglial cells, respectively). Most gliomas are astrocytic tumors (77.5%) [1]. Glioblastoma is the most aggressive astrocytic tumor of the central nervous system (CNS); it accounts for the majority (58.4%) of gliomas, with overall survival as low as 12–15 months [1].

The Cancer Genome Atlas (TCGA) is an integrative multicenter comprehensive project aiming to collect and publish clinically annotated omics profiles for 33 human cancer types; its data are publicly available through the NCI Genomic Data Commons portal (https://portal.gdc.cancer.gov/, accessed on 21 December 2022). Integrated TCGA data collections include clinical information, whole exome sequencing, copy number variation, DNA methylation, gene expression, and protein expression profiles for thousands of individual tumors. This source of multi-omics data is widely used for studying the molecular mechanisms of carcinogenesis and for the discovery and validation (diagnostic, prognostic, and predictive) of cancer biomarkers.

In TCGA, the highest number of samples were collected for the gliomas. Due to considerable molecular and clinical differences between pediatric and adult gliomas [2], pediatric tumors were excluded from TCGA biosampling. TCGA-GBM collection includes a cohort of 606 newly diagnosed grade IV glioblastoma cases (https://portal.gdc.cancer.gov/projects/TCGA-GBM; accessed on 21 December 2022) [3,4]. In TCGA-LGG group, a data collection for 516 newly diagnosed grade II and III brain tumors is deposited (https://portal.gdc.cancer.gov/projects/TCGA-LGG, accessed on 21 December 2022) [5].

TCGA-GBM includes glioblastoma samples collected between 1989 and 2011; 414 patients (76%) were diagnosed in or after 2002 [3,4]. TCGA-LGG collection includes astrocytoma, oligoastrocytoma, and oligodendroglioma samples, 86% of which were collected between 2005 and 2013, and the rest before 2005 [5]. Obviously, all these cases were classified and graded according to the obsolete WHO guidelines [6,7] based solely on histological criteria. The recent 5th edition of the WHO Classification of tumors of the CNS (WHO CNS5) that was published in 2021 introduced numerous changes in classification, diagnostic criteria, nomenclature, and grading of diffuse gliomas. These changes are mostly based on the recommendations of the Consortium to Inform Molecular and Practical Approaches to CNS Tumor Taxonomy (cIMPACT-NOW) [8,9,10,11,12,13,14,15,16,17] and are summarized in [18].

**Figure 1:**
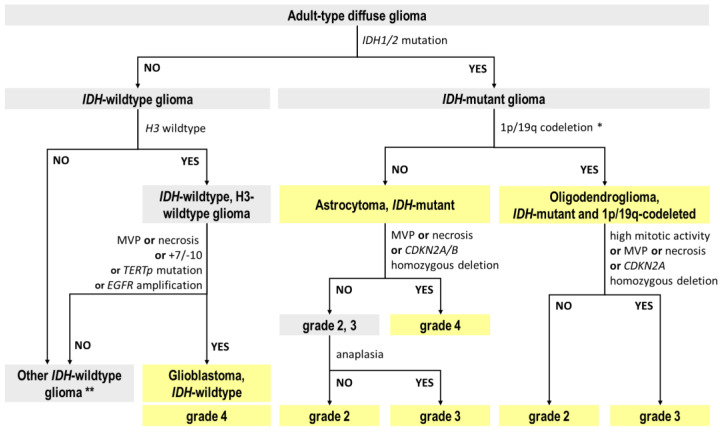
Algorithm of the classification of diffuse gliomas according to the 5th edition of the WHO Classification of tumors of the CNS (WHO CNS5). MVP—microvascular proliferation. * *ATRX* mutation in an *IDH*-mutant diffuse glioma is sufficient for the diagnosis of *IDH*-mutant astrocytoma, obviating the need for 1p/19q testing in order to exclude oligodendroglioma. ** For *IDH*-wildtype gliomas without molecular features of a glioblastoma or when they are unknown it is worth performing a differential diagnosis from pediatric-type diffuse gliomas, especially in the case of young adults.

The WHO CNS5 principles of how molecular features affect the typing and staging of adult-type diffuse gliomas are illustrated in Figure 1. The molecular characteristics and clinical utility of these and other glioma biomarkers were reviewed elsewhere [19].

As an obvious consequence of molecular biomarker implementation in the WHO CNS classification, the diagnosis must change for many previously diagnosed glioma cases, including those from the TCGA-LGG and TCGA-GBM databases. For molecular biology investigations and for the search and validation of new diagnostic, prognostic, and predictive biomarkers, the correct classification of TCGA tumor samples may be necessary. Owing to versatile molecular information about the TCGA samples, such a reclassification is possible in most cases, as is demonstrated by the present study.

Unfortunately, this was not the case for the Chinese Glioma Genome Atlas (CGGA), another large-scale databank of human glioma molecular profiles [20]. In this case molecular data were not sufficient to support reclassification according to the WHO CNS5 rules, thus excluding CGGA profiles from the analysis.

In the current study, for the first time we re-classified available TCGA glioma cases according to the WHO CNS5 guidelines. We also found better correlation between the updated diagnoses and the clinical, epigenomic, and transcriptomic features of the TCGA gliomas.

## 2. Results

### 2.1. Reclassification of TCGA Adult-Type Glioma Cases

TCGA-LGG and TCGA-GBM glioma cases (n = 1122) were initially diagnosed according to the 2000 and 2007 WHO criteria [6,7] based solely on histopathological features. Overall, TCGA gliomas were initially classified into four types: oligodendrogliomas (grades II-III; n = 174), oligoastrocytomas (grades II–III; n = 114), astrocytomas (grades II–III; n = 169), and glioblastomas (grade IV; n = 590). The remaining 75 cases were of unknown histology and, therefore, remained unclassified.

**Figure 2:**
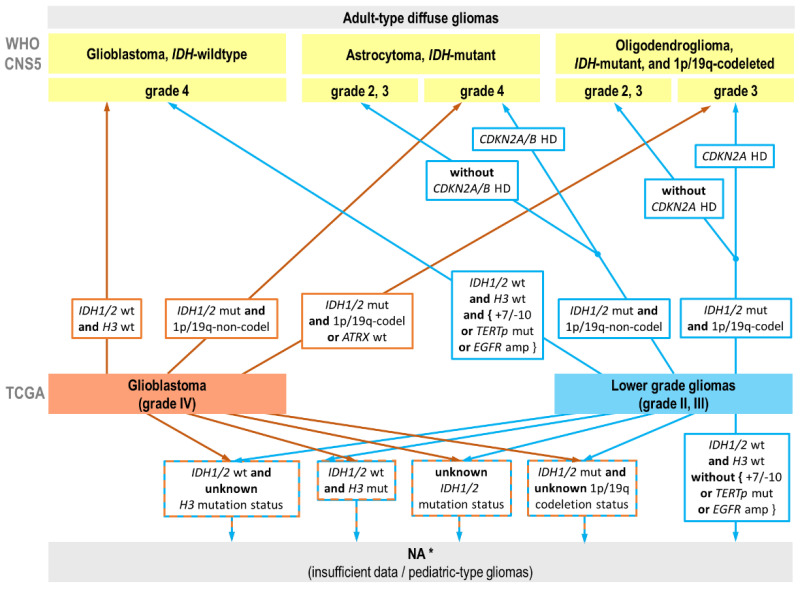
The algorithm of the reclassification of gliomas from The Cancer Genome Atlas (TCGA) databases based on molecular alterations according to WHO CNS5 guidelines. HD—homozygous deletion; wt—wildtype; mut—mutation; amp—amplification. * We combined “NOS” (Not Otherwise Specified) and “NEC” (Not Elsewhere Classified) into «NA» category.

In this study, we extracted the available molecular information to reclassify TCGA glioma cases according to the WHO CNS5 criteria. The algorithm for reclassification of TCGA glioma cohorts according to the WHO CNS5 guidelines is visualized in Figure 2 and summarized in more detail in Table S1.

It is worth noting that in the fifth edition of WHO Blue Books the grade designation has changed from the traditionally used Roman to Arabic numerals. Therefore, throughout the text, Roman and Arabic numerals are used to distinguish the old and the revised WHO CNS tumor nomenclature (WHO CNS5).

The presence of *IDH1/2* mutation is the major molecular diagnostic criterion for the classification of gliomas. In the TCGA annotation, the *IDH1/2* mutation status was unknown for 119 glioblastomas, one oligodendroglioma, and one astrocytoma case. For such samples, we analyzed Mutect2 MAF data from the GDC Data Portal to establish the status for this type of mutation. *IDH1* missense mutation in codon 132 was confirmed for the astrocytoma and oligodendroglioma cases, and *IDH*-wildtype status was confirmed for 13 glioblastomas (Table S2). No genomic data was available for the remaining 106 glioblastomas, so it was not possible to precisely classify them as they could have belonged to either «glioblastoma, *IDH*-wildtype» (WHO grade 4), or «astrocytoma, *IDH*-mutant» (WHO grade 4) types.

According to the WHO CNS5 definition of “glioblastoma, *IDH*-wildtype” as a diffuse, astrocytic glioma that is *IDH*-wildtype and *H3*-wildtype, all *IDH*-wildtype gliomas (n = 551) were checked for *H3* K27 and G34 mutations. Most *IDH*-wildtype gliomas (n = 463) were also *H3*-wildtype, for 85 gliomas there was no genomic data, and in three cases mutation of *H3* was detected. Two patients (age of diagnosis 21 and 30 years) had *H3* G34R substitution and one patient (age of diagnosis 32 years) had *H3* K27M mutation. Younger age and *H3* mutations favor pediatric-type high-grade *H3*-mutant gliomas, namely “*H3* G34–mutant diffuse hemispheric glioma” and “*H3* K27–altered diffuse midline glioma”.

**Figure 3:**
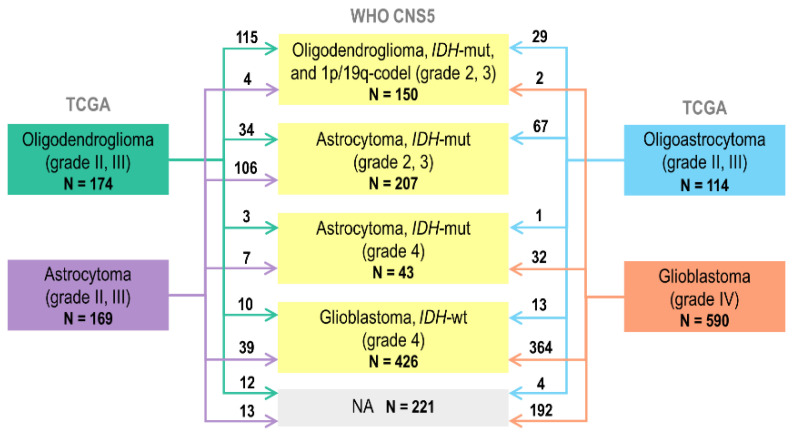
Distribution of TCGA glioma cases according to the WHO CNS5 criteria. Only cases with known histology reviewed (n = 1047).

As a result, 828 cases were reclassified out of 1047 TCGA gliomas analyzed (Figure 3, Table S2). A total of 219 cases fell into the category of «NA» diagnosis formed by two major groups: (1) glioblastomas with unknown *IDH1/2* mutation status (n = 106); and (2) *IDH*-wildtype gliomas with unknown *H3* mutation status (n = 82).

### 2.2. Glioma Clustering with Gene Expression and DNA Methylation Data

We performed unsupervised k-means consensus clustering of TCGA glioma transcriptomic (n = 634) and DNA methylation data (n = 601) in order to check how molecular clusters match with the tumor types according to the “old” (TCGA; WHO CNS3-4) and “new” (WHO CNS5) classifications. Cases with unknown histology were excluded from the analysis.

**Figure 4:**
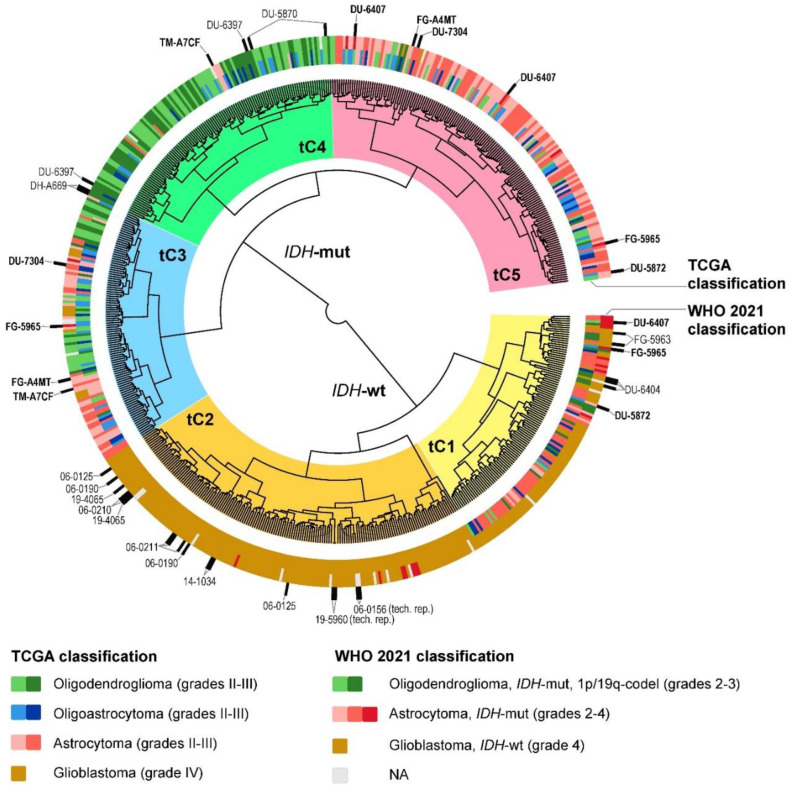
Hierarchical unsupervised clustering dendrogram of RNA sequencing profiles for glioma samples (combined TCGA-LGG and TCGA-GBM databases). Color markers indicate tumor labels according to TCGA clinical annotation, or according to the WHO CNS5 criteria. Clinical cases with two or more transcriptome profiles are highlighted (samples that fall into different clusters are additionally highlighted in bold). tC1–5—transcriptome clusters 1–5. NA—diagnosis is not available due to insufficient data about molecular features of a tumor.

First, we analyzed the transcriptomic subtypes of gliomas (Figure 4, Table S3). The gliomas were divided into two core clusters on a dendrogram consisting mainly of *IDH*-wildtype or *IDH*-mutant tumors (Figure 4). In cluster tC1, the majority of astrocytomas and oligoastrocytomas during reclassification moved to the category of “glioblastoma, *IDH*-wildtype”, while a few cases moved into the category of WHO grade 4 “astrocytoma, *IDH*-mutant”. Notably, WHO grade 4 “astrocytoma, *IDH*-mutant” cases preferentially cluster with WHO grade 4 “glioblastoma, *IDH*-wildtype” rather than WHO grade 2–3 “astrocytoma, *IDH*-mutant”. Cluster tC2 includes almost exclusively “glioblastomas, *IDH*-wildtype” and has undergone only minor changes—namely, a few cases have moved into the category of WHO grade 4 “astrocytoma, *IDH*-mutant”. Clusters tC4 and tC5 became considerably more homogeneous in terms of tumor type after reclassification. Cluster tC4 consists mainly of WHO CNS5 «oligodendroglioma, *IDH*-mutant, and 1p/19q-codeleted», while cluster tC5 consists mainly of «astrocytoma, *IDH*-mutant». The most heterogeneous is cluster tC3, which includes literally all types of tumors. Overall, we found that unsupervised transcriptome clusters are in much closer agreement with our update according to the WHO CNS5 classification rather than with the basic TCGA classification (Figure 4).

Several TCGA glioma cases (n = 18) have two or three transcriptome profiles in the database for different tissue aliquots (marked by black labels on Figure 4). Sometimes such repeats fall into different gene expression clusters (highlighted in bold on Figure 4). This is not surprising, since high intratumoral heterogeneity has been repeatedly documented for the gliomas [21,22,23,24,25,26].

**Figure 5:**
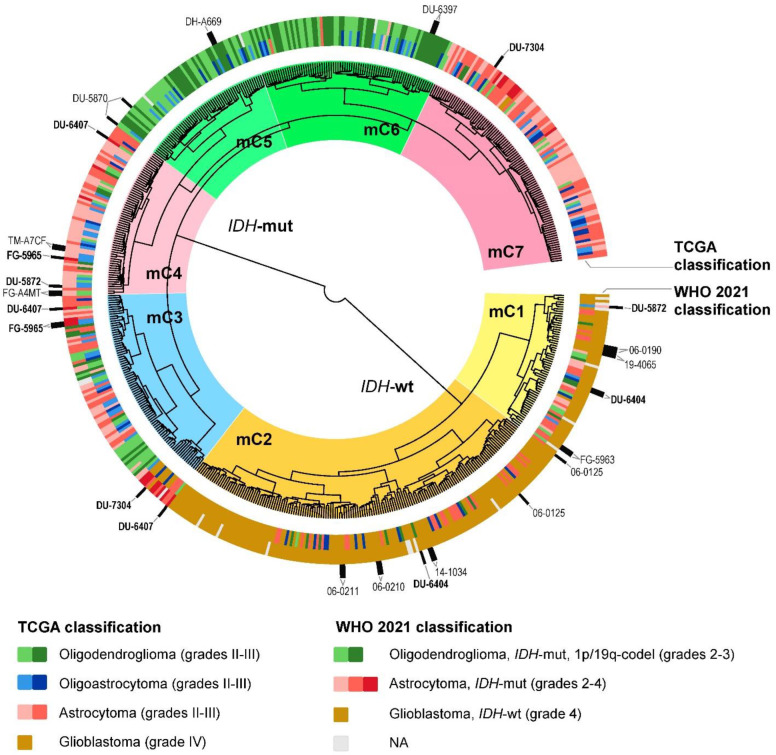
Hierarchical unsupervised clustering dendrogram of DNA methylation profiles for glioma samples (combined TCGA-LGG and TCGA-GBM databases). Color markers indicate tumor labels according to TCGA clinical annotation, or according to the WHO CNS5 criteria. Clinical cases with two or more DNA methylation profiles are highlighted (samples that fall into different clusters are additionally highlighted in bold). mC1–7—DNA methylation clusters 1–7. NA—diagnosis is not available due to insufficient data about molecular features of a tumor.

Then we analyzed the congruence of DNA methylation subtypes and tumor types (Figure 5, Table S4). We aggregated all methylation sites for each gene and performed clustering analysis at the level of gene methylation. Similarly to the transcriptomic clustering, DNA methylation subtypes were tightly connected with the *IDH* mutation status. Most of the other changes for older/newer classification of the gliomas repeated those observed for the gene expression clustering, with the exception that both “astrocytomas, *IDH*-mutant” and «oligodendrogliomas, *IDH*-mutant, and 1p/19q-codeleted» formed not one, but two clusters (mC4/mC7 and mC5/mC6, respectively) and that grade 4 “astrocytomas, *IDH*-mutant” were not clustered together with “glioblastomas, *IDH*-wildtype” (Figure 5).

Finally, the analysis of glioblastoma molecular subtypes was performed. There are several classifications of glioblastoma subtypes, but the most widely used is the one proposed by Dr. Verhaak et al. Initially, his classification included four subtypes (Proneural, Neural, Mesenchymal, and Classical) [27], but it was later updated to three molecular subtypes (Proneural, Mesenchymal, and Classical) [28]. The previously distinguished Neural subtype was identified as normal neural lineage contamination [28].

**Figure 6:**
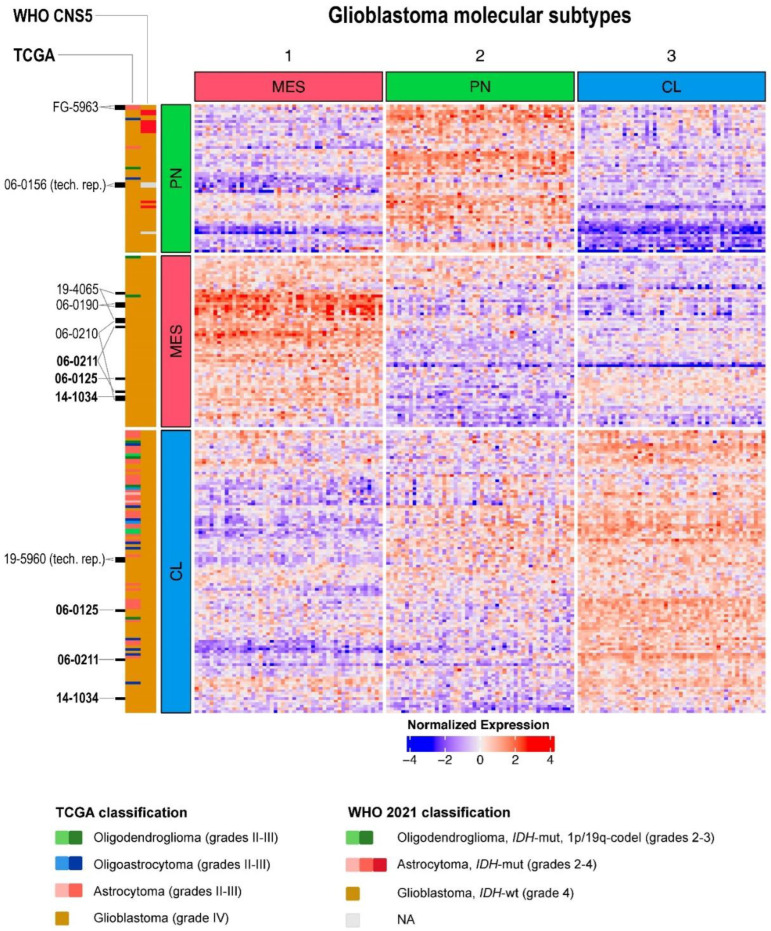
Heatmap of 50-gene signatures for three glioblastoma subtypes [28]. Transcriptomic profiles for glioblastoma samples were taken from combined TCGA-LGG and TCGA-GBM databases. Color markers indicate tumor labels according to TCGA clinical annotation, or according to the WHO CNS5 criteria. Clinical cases with two or more transcriptome profiles are highlighted (samples that fall into different clusters are additionally highlighted in bold). MES—Mesenchymal subtype; PN—Proneural subtype; CL—Classical subtype; NA—diagnosis is not available due to insufficient data about molecular features of a tumor.

We used the updated 150-gene signature [28] to check how transcriptome subtypes of glioblastoma matched with the new WHO glioma classification. Only those cases (n = 222) that were either annotated as glioblastomas in TCGA database or identified by us as “glioblastomas, *IDH*-wildtype” according to the WHO CNS5 criteria were analyzed. The Mesenchymal cluster (n = 66) remained almost unchanged, while Proneural and Classical have changed drastically (Figure 6, Table S5). In the Classical cluster, which consists uniformly of WHO CNS5 “glioblastomas, *IDH*-wildtype”, half of the cases (55 out of 108 tumors) were “molecular glioblastomas” (i.e., histologically lower-grade gliomas but with the molecular features of a glioblastoma), which were previously classified as grade II-III astrocytomas, oligoastrocytomas, and oligodendrogliomas. Newly introduced grade 4 “astrocytomas, *IDH*-mutant” were localized only in the Proneural cluster. As in the case of whole transcriptome unsupervised analysis (Figure 4, Table S3), some tumor aliquots fell into different expression clusters (highlighted in bold on Figure 6), confirming the known intratumoral heterogeneity of glioblastomas.

Thus, based on clustering features with both types of molecular data, we conclude that the new 2021 WHO classification of CNS tumors significantly better reflects the “natural” molecular subtypes of diffuse gliomas compared to the basic TCGA clinical annotation of the samples.

### 2.3. Prognostic Value of the WHO CNS5 Glioma Classification

Our next step was to analyze overall survival (OS) by Kaplan–Meier and Cox regression both for the basic TCGA glioma cohorts («astrocytoma», «oligoastrocytoma», «oligodendroglioma», «glioblastoma») and for the revised cohorts formed in accordance with the WHO CNS5 classification («astrocytoma, *IDH*-mutant», «oligodendroglioma, *IDH*-mutant, and 1p/19q-codeleted», «glioblastoma, *IDH*-wildtype»). The goal was to check how “old” and “new” glioma types reflect patient survival records. Only samples with survival data were used in the analysis (n = 1043).

**Figure 7:**
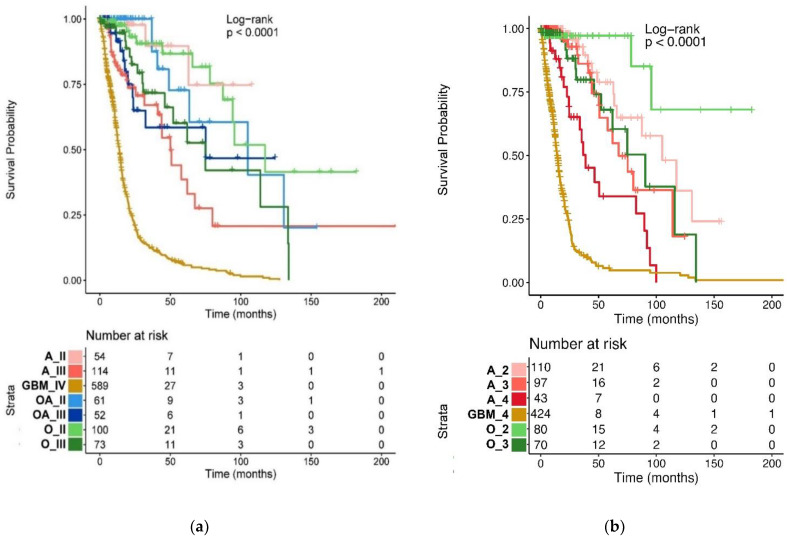
Kaplan–Meier analysis of 1043 TCGA patients with gliomas. Patient groups are shown according to the basic TCGA (**a**) and the WHO CNS5 (**b**) classifications. A_II-III—astrocytoma, grade II–III; GBM_IV—glioblastoma, grade IV; OA_II-III—oligoastrocytoma, grade II–III; O_II-III—oligodendroglioma, grade II–III; GBM_4—glioblastoma, *IDH*-wildtype, grade 4; O_2-3—oligodendroglioma, *IDH*-mutant, and 1p/19q-codeleted, grade 2–3; A_2-4—astrocytoma, *IDH*-mutant, grade 2–4.

The resulting Kaplan–Meier survival curves are shown in Figure 7. Median OS for patient cohorts is listed in Table 1.

**Figure 8:**
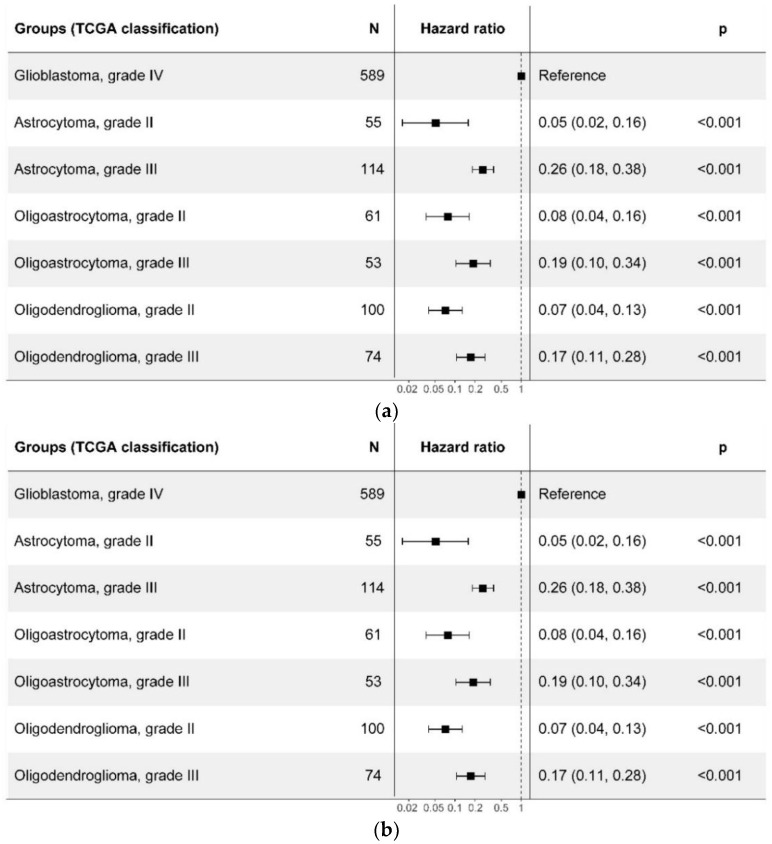
Forest plot of univariate hazard ratio of overall survival for adult patients with diffuse gliomas. Patient groups are shown according to the basic TCGA (**a**) and the WHO CNS5 (**b**) classifications.

As the median OS for glioblastomas did not change after reclassification (13.9 (95% CI: 12.6, 14.9) vs 14.0 (95% CI: 12.7, 15.6) months), this cohort was selected as a reference group for Cox proportional hazards regression (Figure 8). In the case of basic TCGA classification, patients with grade II and grade III gliomas had a similar median OS (Table 1) and hazard ratio (HR) regardless of tumor type (Figure 8A). That is, with the basic classification, glioma prognosis depended mostly on the histopathological grade, not on the tumor type. However, when classified after the WHO CNS5, differences between grade 2 tumor types became much more significant: while patients with “oligodendroglioma, grade II” and “astrocytoma, grade II” had very close HR in comparison to “glioblastoma, grade IV” (HR 0.07 and 0.05, respectively; all for the basic TCGA classification), for the WHO CNS5 classification the risk for patients with “oligodendroglioma, *IDH*-mutant, and 1p/19q-codeleted, grade 2” was 2.5 times lower than for patients with “astrocytomas, *IDH*-mutant, grade 2” (HR 0.03 and 0.08, respectively, in comparison to “glioblastoma, *IDH*-wildtype”) B and Figure 8B). Simultaneously, survival prognosis for grade 3 *IDH*-mutant gliomas was the same for both classifications. Despite perturbations in the group of glioblastomas (Figure 2 and Figure 3), the survival prognosis for the category of “glioblastoma, *IDH*-wildtype, grade 4” remained virtually unchanged compared to “glioblastoma, *IDH*-wildtype, grade IV” (Table 1, Figure 7). This is most probably due to the fact that TCGA sampling included mainly the primary glioblastoma cases, i.e., *IDH1/2* wildtype. Otherwise, the changes could be noticeable. The newly recognized category of «astrocytoma, *IDH*-mutant, grade 4», separated from the basic category “glioblastoma, grade IV”, had a three times better prognosis compared to “glioblastoma, *IDH*-wildtype, grade 4” (HR = 0.32; 95% CI: 0.21, 0.51; *p* < 0.001).

**Figure 9:**
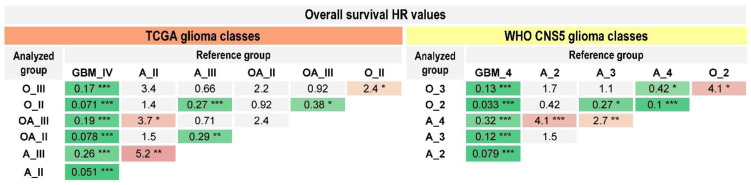
Pairwise comparisons for overall survival for patients with diffuse gliomas according to the basic TCGA and the WHO CNS5 classifications. CI—confidence interval; HR—hazard ratio. GBM_IV—glioblastoma, grade IV; OA_II-III—oligoastrocytoma, grade II–III; O_II-III—oligodendroglioma, grade II–III; A_II-III—astrocytoma, grades II–III; GBM_4—glioblastoma, *IDH*-wildtype, grade 4; O_2-3—oligodendroglioma, *IDH*-mutant, and 1p/19q-codeleted, grade 2–3; A_2-4—astrocytoma, *IDH*-mutant, grade 2–4. * *p* ≤ 0.05; ** *p* ≤ 0.01; *** *p* ≤ 0.001.

To check the statistical significance of differences in OS within tumor types or tumor grades we performed pairwise comparison for groups from the “old” and “new” classifications (Figure 9, Table S6). As discussed above, the prognosis for basic TCGA types of gliomas with the same grade was not statistically different. The same phenomenon was observed for the comparisons of WHO CNS5 diffuse gliomas of the same grade. In particular, “astrocytoma, *IDH*-mutant, grade 3” and “oligodendroglioma, *IDH*-mutant, and 1p/19q-codeleted, grade 3” were indistinguishable in terms of survival prognosis. The difference between the OS of “astrocytoma, *IDH*-mutant, grade 2” and that of “oligodendroglioma, *IDH*-mutant, and 1p/19q-codeleted, grade 2” was also not statistically significant. In contrast, the differences in OS were more pronounced for grade 3 vs grade 2 oligodendrogliomas (HR = 2.4 for the basic TCGA classification, and 4.1 for the WHO CNS5 classification) (Figure 9, Table S6).

As an indicator of prognosis value, we estimated the proportion of statistically significant differences in pairwise comparisons made for the “old” and “new” glioma classes, which was 57% (12/21) for the basic TCGA groups and 73% (11/15) for the WHO CNS5 types (Figure 9).

Finally, we conclude that, on the available TCGA sampling, the integrated WHO CNS5 classification, which considers molecular features of gliomas, generally better serves to distinguish between the survival prognosis for the different groups, as compared to the outdated basic classification.

### 2.4. Performance of Previously Published Glioma Transcriptional Biomarkers

Glioma management still lacks suitable diagnostic, predictive, and prognostic biomarkers. Nonetheless, a number of diagnostic and prognostic biomarkers were proposed [29]. In particular, it had been previously shown that high expression of genes *FREM2* (FRAS1 Related Extracellular Matrix 2) and *SPRY1* (Sprouty RTK Signaling Antagonist 1) is specific to glioblastomas [29,30,31]. Lower *FREM2* expression and overexpression of *CRNDE* (Colorectal Neoplasia Differentially Expressed) non-coding RNA are associated with worse prognosis for glioblastoma patients [30,32]. We had earlier reconstructed FREM2 molecular pathway and tested the FREM2 pathway activation level as a diagnostic and prognostic biomarker. We found that FREM2 pathway was a superior biomarker to the *FREM2* gene expression level itself to discriminate glioblastomas and lower grade gliomas and to predict survival within different glioma subgroups [33]. In addition, a higher FREM2 pathway activation level was associated with lower progression-free survival in gliomas [33]. Many prognostic gene signatures for glioblastomas have been proposed in recent years. For example, in a very recent work [34], a risk model based on the four stiffness-related signature genes (*FN1*, *ITGA5*, *OSMR*, and *NGFR*) was proposed.

All the above-mentioned biomarkers were developed on the basis of the outdated WHO classification of CNS tumors. In this study we aimed to interrogate their efficacy for the WHO CNS5-classified gliomas and to answer the question of whether reconsidering the previous biomarkers may be necessary for the new, WHO CNS5-based glioma diagnoses.

To this end, we tested the performance of the above biomarkers for the revised groups of TCGA gliomas. We investigated how the expression levels of *CRNDE*, *FREM2*, and *SPRY1* genes were connected with the “old” and “new” tumor types. In addition, three variants of the FREM2 pathway with sequentially interacting nodes for one, two, or three levels of interactions were reconstructed as previously described [33]. The first variant (depth = 1) includes 4 nodes, 10 edges, and 53 gene products; the second variant (depth = 2) has 12 nodes, 26 edges, and 69 genes; the third variant (depth = 3) has 66 nodes, 147 edges, and 208 genes [33]. Activator/repressor roles were algorithmically calculated for every gene product according to [35]. The metric of pathway activation level (PAL) quantitatively reflects activation or inhibition of a pathway [36,37]. The area under the ROC curve (AUC) metric was used to quantitatively assess the performance of all above gene- and pathway-based biomarkers.

**Figure 10:**
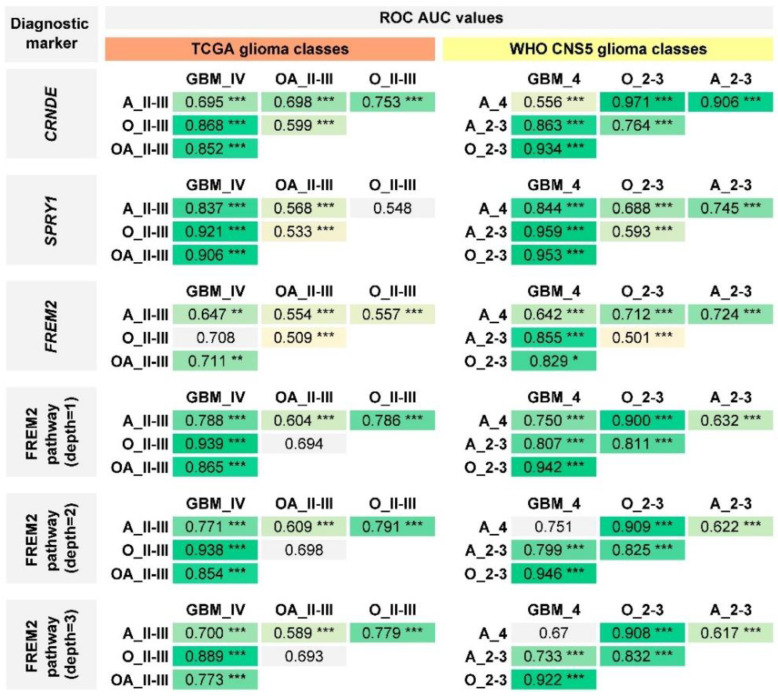
Performance of *CRNDE*, *SPRY1*, and *FREM2* expression and FREM2 pathway activation levels for discrimination of glioma types. GBM_IV—glioblastoma, grade IV (n = 169); OA_II-III—oligoastrocytoma, grade II–III (n = 115); O_II-III—oligodendroglioma, grade II–III (n = 181); A_II-III—astrocytoma, grade II–III (n = 169); GBM_4—glioblastoma, *IDH*-wildtype, grade 4 (n = 220); O_2-3—oligodendroglioma, *IDH*-mutant, and 1p/19q-codeleted, grade 2–3 (n = 150); A_2-3—astrocytoma, *IDH*-mutant, grade 2–3 (n = 206); A_4—astrocytoma, *IDH*-mutant, grade 4 (n = 24). * *p* ≤ 0.05; ** *p* ≤ 0.01; *** *p* ≤ 0.001.

We found a controversial situation regarding the diagnostic biomarkers under investigation (Figure 10, Table S7). When the “old” classification was used, the expression of *CRNDE*, *FREM2*, and *SPRY* genes allowed the diagnosis of “glioblastoma, grade IV” to be differentiated from the lower grade gliomas (astrocytomas, oligoastrocytomas, and oligodendrogliomas). However, the use of the WHO CNS5 classification has transformed *CRNDE* into the diagnostic biomarker of *IDH*-mutant lower grade gliomas. *FREM2* appeared a relatively weak diagnostic biomarker as it showed low or statistically unreliable AUC values for almost all pairwise comparisons except “glioblastoma, *IDH*-wildtype” vs “oligodendroglioma, *IDH*-mutant, and 1p/19q-codeleted”. Much better results were obtained for the activation of FREM2 pathway, as PALs returned relatively higher AUC values and allowed all major types of gliomas to be distinguished from each other, with the only exception being low- and high-grade “astrocytomas, *IDH*-mutant”. A similar figure was observed for another biomarker under analysis, expression of *SPRY1* gene. At the same time, discussed biomarkers are not equivalent to *IDH1/2* mutation status, since they allow glioma type-specific differential diagnostics within the group of *IDH*-mutant tumors with AUC > 0.7.

**Figure 11:**
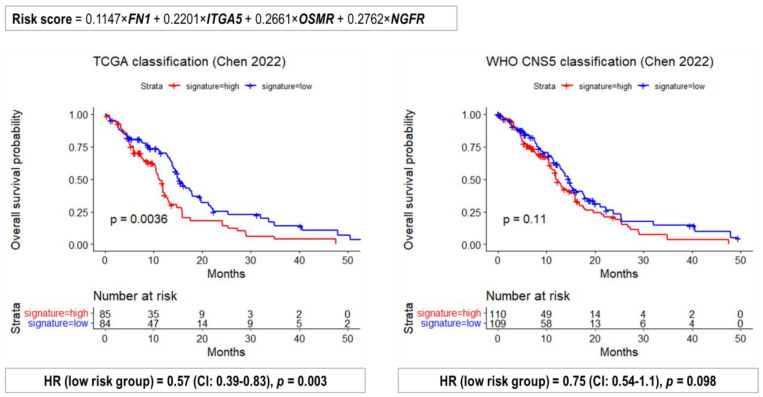
Performance of the 4-gene signature [35] to predict the survival of patients with glioblastoma according to the basic TCGA (**left panel**) and the WHO CNS5 (**right panel**) classifications.

To evaluate the performance of prognostic 4-gene signature risk scores were calculated for each glioblastoma patient (Table S2) according to the original publication [34]. Then Kaplan–Meier plots were drawn and HR was determined for high and low risk groups (Figure 11). The tested prognostic signature showed a dramatic drop in predictive value for the updated “glioblastoma, *IDH*-wildtype” group.

Overall, it can be concluded that the changes introduced by the WHO CNS5 classification of gliomas have dramatically influenced the usability of the previously discovered and validated biomarkers, sometimes making them ineffective. Thus, there is a need to refine the existing pool or to search for the new diagnostic biomarkers of human gliomas that would be based on an updated molecular diagnosis, such as the update to the TCGA glioma database communicated in this report.

## 3. Discussion

Due to the large-scale versatile clinical and multi-omics molecular data for human cancer cases including gliomas, TCGA is now a premium open access database for molecular biomedical studies. However, as it is currently on the project web site, “TCGA-LGG” and “TCGA-GBM” databases dedicated to human gliomas contain obsolete diagnosis annotations based solely on histological laboratory assessments. Since the correct categorization of published glioma cases is crucial for the correct molecular investigations, including the search for new biomarkers, we reclassified the available TCGA glioma cases to make the diagnosis consistent with the current WHO classification of central nervous system tumors (WHO CNS5), which also includes the interrogation of molecular data such as diagnostic mutations and chromosomal rearrangements. This was not possible for the CGGA database, another large collection of molecular data on human gliomas, because of insufficient annotation of the profiles in respect to the diagnostic mutations and rearrangements.

We also tested the way these changes in the groups of gliomas were related to molecular clusters (based on transcriptomic or DNA methylation profiles) and found an overall better agreement with the WHO CNS5 tumor types than with the basic TCGA classification. The evaluation of glioblastoma case distribution among distinct molecular subtypes (Proneural, Mesenchymal, and Classical) showed that “molecular glioblastomas” (i.e., histologically lower-grade gliomas but with the molecular features of a glioblastoma) belong distinctly to the Classical glioblastoma-intrinsic transcriptional subtype.

Overall, our results showed that molecular marker-based diagnosis provides a more objective determination of glioma type. However, several “bottlenecks” are still present, e.g., that profiles for different tissue aliquots may fall into different molecular clusters, which is related to the well-known intratumoral heterogeneity in CNS tumors, especially in the glioblastomas [21,26]. Furthermore, not all cases were well separated into glioma types by unsupervised clustering analysis, forming mixed clusters, which requires further study.

Our next goal was to check the survival prognosis performance of the new glioma classification using TCGA data. We found that the “new” (WHO CNS5) tumor types generally more accurately reflect survival estimates compared with the “old” ones which were based on histological diagnosis alone. Better survival stratification increases the likelihood of discovering new effective prognostic biomarkers.

In addition, we tested several previously established and published diagnostic and prognostic biomarkers to investigate whether reclassification according to the WHO CNS5 has affected their clinical relevance. All examined single genes, gene signatures, and molecular pathway-based expression biomarkers behaved differently for the older and newer glioma classes. Some of the previously established gene expression-based molecular markers that performed well for the “old” glioma classes could be of little value for the updated groups of the gliomas. This suggests that the previous studies including high-throughput biomarker investigations based on TCGA, CGGA [38], and other sources of available information including experimental cohorts [39,40,41], and their results may become out-of-date.

Another important conclusion is that previous secondary molecular databanks that aggregate published molecular and clinical data including patient diagnosis and response on treatment must be reconsidered as well [42]. Finally, different classification of patients can also compromise the previous results obtained for the proteomic data [43] and for the comparison of transcriptomic and proteomic [38] data.

Thus, we claim that there is a need to search for and to validate new diagnostic biomarkers for diffuse gliomas that would be consistent with the latest WHO CNS5 classification.

Although our findings don’t have a direct clinical impact, it can be considered of high importance for many applied studies aimed at developing molecular biomarkers for gliomas, because molecular criteria of previous (WHO CNS3-4) and updated (WHO CNS5) glioma classes differ dramatically. Our results also show that the changes occurred in the glioma classification cannot be ignored when comparing and interpreting the results of clinical trials conducted before and after the introduction of the WHO CNS5, since the clinical features of gliomas, such as survival prognosis, have also changed.

## 4. Materials and Methods

### 4.1. The Cancer Genome Atlas (TCGA) Data

There are a total of 1122 glioma cases in TCGA-LGG and TCGA-GBM datasets. Cases with unknown histology (n = 75) were excluded from the analysis. *IDH1/2*, *ATRX*, and *TERTp* mutation statuses, *EGFR* amplification, gain of chromosome 7 and loss of chromosome 10, and 1p/19q-codeletion were taken from [44]. In the TCGA annotation, *IDH1/2* mutation status was unknown for 121 gliomas. For such samples, we analyzed Mutect2 MAF data from the GDC Data Portal to establish the status for this type of mutation. Additionally, mutation in histone H3 subgroup has been determined: specifically, mutations p.Lys28Met (K27M) in genes *H3-3A* (*H3F3A*), *H3C2* (*HIST1H3B*), and *H3C3* (*HIST1H3C*) and p.Gly35Arg (G34R) in gene *H3-3A*. *EGFR* amplification, *CDKN2A/B* deletion were extracted from corresponding CNV files from the GDC Data Portal (https://portal.gdc.cancer.gov/repository, accessed on 9 November 2022) [45]. RNA-seq data (HTseq counts) and DNA methylation array data (SeSAMe methylation beta estimations [46]) were downloaded from the GDC Data Portal (https://portal.gdc.cancer.gov/, accessed on 1 August 2022) [45]. RNA-seq data were normalized using DESeq2 [47]. Overall survival data were extracted from clinical annotations on the GDC Data Portal for 589 TCGA-GBM and 457 TCGA-LGG samples with known histology [45].

### 4.2. Transcriptional Glioblastoma Subtypes

The clustering of transcriptional profiles based on tumor-intrinsic gene signatures from Verhaak et al. [28] was conducted on Z-scores of RPKM values, which then were converted to a log2 scale according to the original article. Hierarchical clustering was performed with “ward.D2”. R build-in native statistical methods were used to perform all the statistical analysis.4.3. Pathway Activation Level Calculation

### 4.3. Pathway Activation Level Calculation

Pathway activation level (PAL) is an integral quantitative and qualitative characteristic of changes in the expression levels of genes participating in a certain molecular pathway [37]. PALs were calculated as follows:

$$PAL_{p}=\underset{n}{\sum }ARR_{np}\times \log_{10}(CNR_{n})\times 100\;÷\;\underset{n}{\sum }\left|ARR_{np}\right|,\;$$

where *PAL_p_* is PAL for pathway *p*; *CNR_n_* is case-to-normal ratio, the ratio of gene n expression in a sample under study to an average level in the control group; and *ARR* (activator/repressor role) is a value that depends on the function of this gene product in pathway *p*. ARRs are values defined as follows: −1 when product of gene *n* inhibits *p*; 1 when product of n activates *p*; 0 when product of n has an ambiguous or unclear role in the pathway; and 0.5 or −0.5, when the product of n is an activator or an inhibitor of *p*, respectively. As the reference gene expression profile we used the artificial gene expression profile obtained by gene-by-gene averaging of all gene expression data in the cohort under investigation.

FREM2 pathway was reconstructed as described by Zolotovskaia M. et al. [33].

### 4.4. Statistical Analysis

ROC AUC value was used as the measure of biomarker quality. Overall survival was assessed by Kaplan–Meier analysis; the statistical significance of differences was measured by log-rank test *p*-value. Hazard ratios (HR) were calculated in the Cox model to assess differences in survival among the groups under comparison. Cox survival analysis for OS and PFS was performed between those two groups using R packages “survival” v3.2-11 [48] and “survminer” v0.4.9 [49]. Kaplan–Meier plots were drawn using “ggsurvplot” (“survminer” v0.4.9) R function [49]. Tables with hazard ratio, confidence intervals, and p-values were drawn using “ggforest” (“survminer” v0.4.9) R function [49]. Hierarchical clustering was performed using “ward.d2” method in R. Dendrogram was visualized using “dendextend” v0.1.23 R package [50].

## Figures and Tables

**Table 1:** Median overall survival for TCGA-LGG and TCGA-GBM diffuse gliomas before (upper panel) and after (bottom panel) the classification according to the WHO CNS5 criteria.

TCGA Glioma Types	Median OS (95% CI), Months
Oligodendroglioma, grade II (n = 100)	117.3 (94.5-NA)
Oligodendroglioma, grade III (n = 73)	75 (52.1-NA)
Oligoastrocytoma, grade II (n = 61)	105.1 (63.5-NA)
Oligoastrocytoma, grade III (n = 52)	75.1 (23.7-NA)
Astrocytoma, grade II (n = 54)	NA
Astrocytoma, grade III (n = 114)	50.1 (43.9-NA)
Glioblastoma, grade IV (n = 589)	13.9 (12.6–14.9)
**WHO CNS5 glioma types**	**Median OS (95% CI), months**
Oligodendroglioma, *IDH*-mutant, and 1p/19q-codeleted, grade 2 (n = 80)	NA (95.5-NA)
Oligodendroglioma, *IDH*-mutant, and 1p/19q-codeleted, grade 3 (n = 70)	90.5 (62-NA)
Astrocytoma, *IDH*-mutant, grade 2 (n = 110)	105.1 (65.7-NA)
Astrocytoma, *IDH*-mutant, grade 3 (n = 97)	67.4 (50.8-NA)
Astrocytoma, *IDH*-mutant, grade 4 (n = 43)	38.7 (24.6–91.7)
Glioblastoma, *IDH*-wildtype, grade 4 (n = 424)	14.0 (12.7–15.6)

OS—overall survival; CI—confidence interval; NA—not available.

## Data Availability

Not applicable.

## Supplementary Materials

The following supporting information can be downloaded at: https://www.mdpi.com/article/10.3390/ijms24010157/s1.
