# Supplementary material for: Reclassification of TCGA Diffuse Glioma Profiles Linked to Transcriptomic, Epigenetic, Genomic and Clinical Data, According to the 2021 WHO CNS Tumor Classification
Source: Int J Mol Sci. 2022 Dec 21;24(1):157. doi: 10.3390/ijms24010157 (PMC9820617; doi:10.3390/ijms24010157)
Supplement: Supplementary file 1 [file ijms-24-00157-s001.zip › ijms-2057006_Table S1.pdf]

Table S1. Criteria for reclassification of TCGA diffuse gliomas in accordance with the up-to-date WHO classification of CNS tumors (WHO CNS5)

TCGA classification	TCGA grade	Molecular markers				WHO CNS5 glioma type *	WHO CNS5 grade *	Category	Number of cases	Comments
Oligodendroglioma **N=174**	Grade II	*IDH1/2*-mutant	1p/19q-codeleted	**without** *ATRX* mutation	**without** *CDKN2A* homozygous deletion	**Oligodendroglioma, *IDH*-mutant, and 1p/19q-codeleted**	Grade 2	1.1	**62**	-
	Grade III	*IDH1/2*-mutant	1p/19q-codeleted	**without** *ATRX* mutation	-	**Oligodendroglioma, *IDH*-mutant, and 1p/19q-codeleted**	Grade 3	1.2	**53**	-
	Grade II	*IDH1/2*-mutant	1p/19q-codeleted	**without** *ATRX* mutation	*CDKN2A* homozygous deletion	**Oligodendroglioma, *IDH*-mutant, and 1p/19q-codeleted**	Grade 3	1.3	**0**	-
	Grade II	*IDH1/2*-mutant	1p/19q-codeleted	*ATRX* mutation	-	NA	NA	1.4	**0**	As “oligodendrogliomas, *IDH*-mutant, and 1p/19q-codeleted” lack *ATRX* mutation these cases are left unclassified
	Grade III	*IDH1/2*-mutant	1p/19q-codeleted	*ATRX* mutation	-	NA	NA	1.5	**2**	
	Grade II	*IDH1/2*-mutant	1p/19q-**non**-codeleted	**without** *CDKN2A/B* homozygous deletion	-	**Astrocytoma, *IDH*-mutant**	Grade 2	1.6	**27**	-
	Grade III	*IDH1/2*-mutant	1p/19q-**non**-codeleted	**without** *CDKN2A/B* homozygous deletion	-	**Astrocytoma, *IDH*-mutant**	Grade 3	1.7	**7**	-
	Grade II, III	*IDH1/2*-mutant	1p/19q-**non**-codeleted	*CDKN2A/B* homozygous deletion	-	**Astrocytoma, *IDH*-mutant**	Grade 4	1.8	**3**	-
	Grade II, III	*IDH1/2*-mutant	1p/19q-**non**-codeleted	**unknown** *CDKN2A/B* homozygous deletion status	-	**Astrocytoma, *IDH*-mutant**	Grade NA	1.9	**1**	As CDKN2A/B HD status is unknown, the grade cannot be determined
	Grade II, III	*IDH1/2*-wildtype	+7/-10 **OR** *TERT* promoter mutation	**without** H3 p.K28 (K27) and H3 p.G35 (G34) mutations	-	**Glioblastoma, *IDH*-wildtype**	Grade 4	1.10	**10**	-

TCGA classification	TCGA grade	Molecular markers				WHO CNS5 glioma type *	WHO CNS5 grade *	Category	Number of cases	Comments
			**OR** *EGFR* amplification							
	Grade II	*IDH1/2*-wildtype	**without** H3 p.K28 (K27) and H3 p.G35 (G34) mutations	**without or unknown** +7/-10 **AND** *TERT* promoter mutation **AND** *EGFR* amplification	-	NA	NA	1.11	**5**	Molecular features required to classify a tumor as “glioblastoma, IDH-wildtype” are absent or unknown
	Grade III	*IDH1/2*-wildtype	**without** H3 p.K28 (K27) and H3 p.G35 (G34) mutations	**without or unknown** +7/-10 **AND** *TERT* promoter mutation **AND** *EGFR* amplification	-	NA	NA	1.12	**3**	
	Grade II, III	*IDH1/2*-wildtype	H3 p.K28 (K27) or H3 p.G35 (G34) mutant	-	-	NA	NA	1.13	**1**	**TCGA-HT-7469** has a mutation *H3-3A* p.G35R (G34R). This indicates a pediatric-type high grade glioma (Diffuse hemispheric glioma, H3 G34-mutant)
	Grade II, III	**unknown** *IDH1/2* mutation status	-	-	-	NA	NA	1.14	**0**	-
Astrocytoma **N=169**	Grade II	*IDH1/2*-mutant	1p/19q-**non**-codeleted	**without** *CDKN2A/B* homozygous deletion	-	**Astrocytoma, *IDH*-mutant**	Grade 2	2.1	**43**	-
	Grade III	*IDH1/2*-mutant	1p/19q-**non**-codeleted	**without** *CDKN2A/B* homozygous deletion	-	**Astrocytoma, *IDH*-mutant**	Grade 3	2.2	**63**	-
	Grade II, III	*IDH1/2*-mutant	1p/19q-**non**-codeleted	*CDKN2A/B* homozygous deletion	-	**Astrocytoma, *IDH*-mutant**	Grade 4	2.3	**7**	-

TCGA classification	TCGA grade	Molecular markers				WHO CNS5 glioma type *	WHO CNS5 grade *	Category	Number of cases	Comments
	Grade II	*IDH1/2*-mutant	1p/19q-codeleted	**without** *ATRX* mutation	**without** *CDKN2A* homozygous deletion	**Oligodendroglioma, *IDH*-mutant, and 1p/19q-codeleted**	Grade 2	2.4	**2**	-
	Grade III	*IDH1/2*-mutant	1p/19q-codeleted	**without** *ATRX* mutation	-	**Oligodendroglioma, *IDH*-mutant, and 1p/19q-codeleted**	Grade 3	2.5	**2**	-
	Grade II	*IDH1/2*-mutant	1p/19q-codeleted	**without** *ATRX* mutation	*CDKN2A* homozygous deletion	**Oligodendroglioma, *IDH*-mutant, and 1p/19q-codeleted**	Grade 3	2.6	**0**	-
	Grade II, III	*IDH1/2*-wildtype	+7/-10<br>**OR** *TERT* promoter mutation<br>**OR** *EGFR* amplification	**without** H3 p.K28 (K27) and H3 p.G35 (G34) mutations	-	**Glioblastoma, *IDH*-wildtype**	Grade 4	2.7	**39**	-
	Grade II	*IDH1/2*-wildtype	**without** H3 p.K28 (K27) and H3 p.G35 (G34) mutations	**without or unknown** +7/-10<br>**AND** *TERT* promoter mutation<br>**AND** *EGFR* amplification	-	NA	NA	2.8	**6**	Molecular features required to classify a tumor as “glioblastoma, IDH-wildtype” are absent or unknown
	Grade III	*IDH1/2*-wildtype	**without** H3 p.K28 (K27) and H3 p.G35 (G34) mutations	**without or unknown** +7/-10<br>**AND** *TERT* promoter mutation<br>**AND** *EGFR* amplification	-	NA	NA	2.9	**6**	
	Grade II, III	*IDH1/2*-wildtype	H3 p.K28 (K27) or H3 p.G35 (G34) mutant	-	-	NA	NA	2.10	**1**	**TCGA-TM-A84C** has a mutation *H3-3A* p.K28M (K27M). This indicates a pediatric-type high grade glioma (Diffuse midline glioma, H3 K27–altered)
	Grade II, III	**unknown** *IDH1/2*	-	-	-	NA	NA	2.11	**0**	-

TCGA classification	TCGA grade	Molecular markers				WHO CNS5 glioma type *	WHO CNS5 grade *	Category	Number of cases	Comments
		mutation status								
Oligoastrocytoma **N=114**	Grade II	*IDH1/2*-mutant	1p/19q-**non**-codeleted	**without** *CDKN2A/B* homozygous deletion	-	**Astrocytoma, *IDH*-mutant**	Grade 2	3.1	**40**	-
	Grade III	*IDH1/2*-mutant	1p/19q-**non**-codeleted	**without** *CDKN2A/B* homozygous deletion	-	**Astrocytoma, *IDH*-mutant**	Grade 3	3.2	**27**	-
	Grade II, III	*IDH1/2*-mutant	1p/19q-**non**-codeleted	*CDKN2A/B* homozygous deletion	-	**Astrocytoma, *IDH*-mutant**	Grade 4	3.3	**1**	-
	Grade II, III	*IDH1/2*-mutant	1p/19q-**non**-codeleted	**unknown** *CDKN2A/B* homozygous deletion status	-	**Astrocytoma, *IDH*-mutant**	Grade NA	3.4	**1**	As CDKN2A/B HD status is unknown, the grade cannot be determined
	Grade II	*IDH1/2*-mutant	1p/19q-codeleted	**without** *ATRX* mutation	**without** *CDKN2A* homozygous deletion	**Oligodendroglioma, IDH-mutant, and 1p/19q-codeleted**	Grade 2	3.5	**16**	-
	Grade III	*IDH1/2*-mutant	1p/19q-codeleted	**without** *ATRX* mutation	**without** *CDKN2A* homozygous deletion	**Oligodendroglioma, *IDH*-mutant, and 1p/19q-codeleted**	Grade 3	3.6	**13**	-
	Grade II	*IDH1/2*-mutant	1p/19q-codeleted	**without** *ATRX* mutation	*CDKN2A* homozygous deletion	**Oligodendroglioma, *IDH*-mutant, and 1p/19q-codeleted**	Grade 3	3.7	**0**	-
	Grade II	*IDH1/2*-mutant	1p/19q-codeleted	*ATRX* mutation	-	NA	NA	3.8	**1**	As “oligodendrogliomas, *IDH*-mutant, and 1p/19q-codeleted” lack *ATRX* mutation these cases are left unclassified
	Grade III	*IDH1/2*-mutant	1p/19q-codeleted	*ATRX* mutation	-	NA	NA	3.9	**0**	
	Grade II	*IDH1/2*-wildtype	**without** H3 p.K28 (K27) and H3	**without or unknown**	-	NA	NA	3.10	**0**	Molecular features required to classify a tumor as “glioblastoma,

TCGA classification	TCGA grade	Molecular markers				WHO CNS5 glioma type *	WHO CNS5 grade *	Category	Number of cases	Comments
			p.G35 (G34) mutations	+7/-10 **AND** *TERT* promoter mutation **AND** *EGFR* amplification						*IDH*-wildtype” are absent or unknown
	Grade III	*IDH1/2*-wildtype	**without** H3 p.K28 (K27) and H3 p.G35 (G34) mutations	**without or unknown** +7/-10 **AND** *TERT* promoter mutation **AND** *EGFR* amplification	-	NA	NA	3.11	**2**	
	Grade II, III	*IDH1/2*-wildtype	+7/-10 **OR** *TERT* promoter mutation **OR** *EGFR* amplification	**without** H3 p.K28 (K27) and H3 p.G35 (G34) mutations	-	**Glioblastoma, *IDH*-wildtype**	Grade 4	3.12	**13**	-
Glioblastoma **N=590**	Grade IV	*IDH1/2*-wildtype	+7/-10 **OR** *TERT* promoter mutation **OR** *EGFR* amplification	**without** H3 p.K28 (K27) and H3 p.G35 (G34) mutations	-	**Glioblastoma, *IDH*-wildtype**	Grade 4	4.1	**300**	-
		IDH1/2-wildtype	**without or unknown** +7/-10 **AND** *TERT* promoter mutation **AND** *EGFR* amplification	**without** H3 p.K28 (K27) and H3 p.G35 (G34) mutations	-	**Glioblastoma, *IDH*-wildtype**	Grade 4	4.2	**64**	We accept that there was necrosis and/or MVP in the samples, otherwise they would not fall into the category of “glioblastoma, grade IV”. As necrosis or/and MVP are WHO CNS5 criteria sufficient for classifying an *IDH*-wildtype diffuse glioma as “glioblastoma, *IDH*-wildtype, grade 4”, tumor type was preserved based on histological features.
		IDH1/2-wildtype	H3 p.K28 (K27) or H3	-	-	NA	Grade 4	4.3	**1**	**TCGA-06-A5U0** has a mutation *H3-3A* p.G35R (G34R). This indicates a pediatric-type high

TCGA classification	TCGA grade	Molecular markers				WHO CNS5 glioma type *	WHO CNS5 grade *	Category	Number of cases	Comments
			p.G35 (G34) mutant							grade glioma (Diffuse hemispheric glioma, H3 G34-mutant)
		IDH1/2-wildtype	**unknown** H3 p.K28 (K27) or H3 p.G35 (G34) mutations status	-	-	NA	Grade 4	4.4	**82**	Accordingly to WHO CNS5, Glioblastoma, *IDH*-wildtype, is a diffuse, astrocytic glioma that is *IDH*-wildtype and *H3*-wildtype. As *H3* mutation status is unknown, diagnosis of a glioblastoma cannot be assigned
		IDH1/2-mutant	1p/19q-**non**-codeleted	-	-	**Astrocytoma, *IDH*-mutant**	Grade 4	4.5	**32**	We accept that there was necrosis and/or MVP in the samples, otherwise they would not fall into the category of “glioblastoma, grade IV”. As necrosis or/and MVP are WHO CNS5 criteria sufficient for classifying an *IDH*-mutant astrocytic tumor as “astrocytoma, *IDH*-mutant, grade 4”, tumor grade was preserved based on histological features.
		IDH1/2-mutant	1p/19q-codeleted	-	-	**Oligodendroglioma, *IDH*-mutant, and 1p/19q-codeleted**	Grade 3	4.6	**2**	Molecular features (*IDH*-mutant, 1p/19q-codeleted) match the diagnosis of “oligodendroglioma, *IDH*-mutant, and 1p/19q-codeleted”.<br>We accept that there was necrosis and/or MVP in the samples, otherwise they would not fall into the category of “glioblastoma, grade IV”. Necrosis or/and MVP are WHO CNS5 histological criteria for grade 3 oligodendroglioma.
		IDH1/2-mutant	**unknown** 1p/19q codeletion status	-	-	NA	NA	4.7	**3**	These cases may belong either to “astrocytomas, *IDH*-mutant, grade 4” or to “oligodendroglioma, *IDH*-mutant, and 1p/19q-codeleted, grade 3” depending on 1p/19q codeletion status

TCGA classification	TCGA grade	Molecular markers				WHO CNS5 glioma type *	WHO CNS5 grade *	Category	Number of cases	Comments
		**unknown** *IDH1/2* mutation status	-	-	-	NA	NA	4.8	**106**	-
unknown **N=75**	unknown	-	-	-	-	NA	NA	5.1	**75**	-

* If the necessary diagnostic information is lacking or does not allow for a certain WHO diagnosis (e.g., in the case of a mismatch between clinical, histological and/or molecular features) the WHO CNS5 prescribes to use descriptive diagnosis with "NEC" (Not Elsewhere Classified) or "NOS" (Not Otherwise Specified) addreviations; we combined all such cases under the designation «**NA**».
